# Distribution of the Asiatic golden cat (*Catopuma temminckii*) and variations in its coat morphology in China

**DOI:** 10.1002/ece3.10900

**Published:** 2024-02-07

**Authors:** Fei Duan, Shuyi Zhu, Yuan Wang, Dazhao Song, Xiaoli Shen, Sheng Li

**Affiliations:** ^1^ National Natural History Museum of China Beijing China; ^2^ School of Life Sciences & Institute of Ecology Peking University Beijing China; ^3^ Wildlife Conservation Monitoring Center, National Forestry and Grassland Administration Beijing China; ^4^ Research Institute of Natural Protected Area Chinese Academy of Forestry Beijing China; ^5^ Tibet Autonomous Region Research Institute of Forestry Inventory and Planning Lhasa China; ^6^ Chinese Felid Conservation Alliance Beijing China; ^7^ State Key Laboratory of Vegetation and Environmental Change, Institute of Botany Chinese Academy of Sciences Beijing China

**Keywords:** camera‐trapping, *Catopuma temminckii*, coat morph, range map, small cats, species distribution model

## Abstract

Of the 12 wild felid species found in China, Asiatic golden cat (*Catopuma temminckii*) is one of the least studied species. This medium‐sized cat with a prominently polymorphic coat was once distributed across much of southern China, but is believed to have experienced severe decline and range contraction during the past decades, primarily due to anthropogenic pressures. A lack of knowledge of its current distribution, ecology, and natural history has greatly hindered the implementation of conservation and management actions for this species. In this study, for the first time, we compiled the state‐wide occurrence records (*N* = 409), mainly from the camera‐trapping surveys, of Asiatic golden cats from 2008 to 2019, and predicted its distribution across the country through species distribution modeling using random forest algorithm. The results showed that the predicted habitats were mainly located in southwest China and suggested a rather low probability of possible current distribution across its vast historic range in central, eastern, and southern China. We divided its current range into four geographic regions (i.e., Qinling Mountains, Hengduan Mountains, East Himalayas, and southern Yunnan region) and considered the cats in each region as a regional population within the country. From the 287 camera‐trapping detections with photographs and/or videos collected across all populations, we identified six coat morphs and determined their occurrence percentages: common golden (47.4%), spotted (20.9%), red (13.6%), dark cinnamon (10.1%), melanistic (7.0%), and gray (1.0%). The complexity of coat morph composition within regional populations showed an increasing gradient from northeast to southwest. Among the four regional populations, the East Himalayas hosted the highest abundance and coat variation with all six morphs recorded. Our study results update the current distribution and coat morphology variations of this elusive cat in China and provide important knowledge to guide future research and conservation planning for this threatened species.

## Graphical abstract

In this study, for the first time, we compiled the state‐wide occurrence records (*N* = 409), mainly from the camera‐trapping surveys, of Asiatic golden cats from 2008 to 2019, and predicted its current distribution across the country through species distribution modeling using random forest algorithm. The results showed that the predicted habitats were mainly located in southwest China. From the 287 camera‐trapping detections with photographs and/or videos collected across all populations, we identified six coat morphs and found the complexity of coat morph composition within regional populations showed an increasing gradient from northeast to southwest.

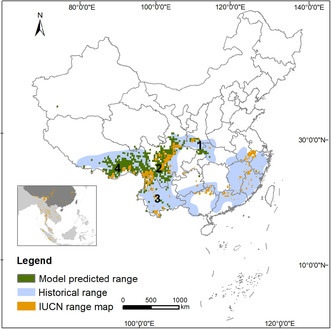

## 1 INTRODUCTION

While China is home to one of the richest feline communities in the world (IUCN/SSC Cat Specialist Group, 2014; Jiang et al., 2017; Wei et al., 2021), most of its research and conservation efforts have focused on a few large, iconic species, such as tigers (*Panthera tigris*), leopards (*P. pardus*), and snow leopards (*P. uncia*) (Li, Weckworth, et al., 2020; Vitkalova et al., 2018; Wang et al., 2016). Small cats, which are more diverse in species but less charming, have received comparatively little attention, and neglect has resulted in a dearth of ecological knowledge of these species, including their distributions, population dynamics, and habitat requirements. That lack of information has greatly hindered the implementation of conservation and management actions for these small cats (IUCN/SSC Cat Specialist Group, 2014).

The Asiatic golden cat (*Catopuma temminckii*), with a body mass of 8–16 kg, is a medium‐sized cat (Sunquist & Sunquist, 2002) that preys on a wide range of species (mainly small vertebrates such as rats, snakes, lizards, birds, and occasionally, larger species such as ungulates; Grassman et al., 2005; Kawanishi & Sunquist, 2008; Xiong et al., 2017). It inhabits a variety of habitat types such as evergreen broadleaf forests, dry deciduous forests, coniferous forests, and shrublands (Nowell & Jackson, 1996; Smith & Xie, 2008). Their distribution spans from lowland rain forests to alpine Rhododendron forests (>3000 m above sea level), and individuals have been recorded as high as about 4300 m (4282 m in Bhutan: Dhendup et al., 2016 and 4290 m in China: Qiao et al., 2022). Records of two radio‐collared individuals from Phu Khieu National Park in Thailand showed that the species is diurnal and crepuscular and individuals occupied fairly large home ranges (32.6–47.7 km^2^) and moved an average of 1597 m per day (Grassman et al., 2005).

Asiatic golden cats were once widely distributed throughout much of tropical and subtropical Asia, from southern and southwestern China to the southern Himalayas and southeastern Asia, including China, Vietnam, Laos, Cambodia, Thailand, Malaysia, Indonesia, Myanmar, India, Bangladesh, Bhutan, and Nepal (Sunquist & Sunquist, 2002; Tempa et al., 2013). However, over the past half century its range has shrunk drastically and its existing habitat is believed highly fragmented (Liu, Wu, et al., 2020; McCarthy et al., 2015). A recent range‐wide assessment by Petersen et al. (2021) indicated a 68% decline in area of this species between 2000 and 2020, with a further 18% decline predicted over the next 20 years. Its historical distribution in China covered most of the eastern, central, southern, and southwestern mainland provinces (Liu, Wu, et al., 2020), but its current distribution is unknown. Habitat loss due to logging and urban/agricultural expansion are considered the major threats to this forest‐dependent species (Choudhury, 2007; McCarthy et al., 2015), but economically driven poaching and harvesting for fur and bone also contribute to their dramatic declines (Willcox et al., 2014). They are also frequently killed as by‐catch in snares set for bushmeat hunting of ungulates (Sunquist & Sunquist, 2002). The cat is classified as Near Threatened by the IUCN Red List of Threatened Species (McCarthy et al., 2015) and is recently upgraded to a Class‐I State Key Protected Species in China.

Among all felid species, the Asiatic golden cat has a particularly notable morphology. Its highly polymorphic pelage comes in various coat colors and patterns (Gao et al., 1987; Liu, Wu, et al., 2020; Wilson & Mittermeier, 2014). The most common coat morphs are a bright red morph found in tropical forests (Ghimirey & Pal, 2009) and a pale sandy morph found in temperate and subtropical regions (e.g., central and southwestern China; Liu, Wu, et al., 2020; Wang et al., 2019). In addition, intermediate forms were also recorded, e.g., the golden form with water marks (Gumal et al., 2014). Occasionally, individuals of other morphs, such as spotted, cinnamon, gray, and melanistic, have been reported (Ghimirey & Pal, 2009; Holden, 2001; Li et al., 2010; Nijhawan et al., 2019). Previous studies have also shown that individuals of different morphs may co‐occur in a region (Nijhawan et al., 2019; Vernes et al., 2015; Wang et al., 2019), but aspects of this phenomenon, such as the proportions of different coat morphs within a specific population or across regional populations, are not well understood.

Over the past two decades, camera‐trapping has been increasingly used for wildlife research and monitoring. It has become one of the most popular and efficient tools to survey large‐ and medium‐sized terrestrial animals, especially carnivores that occur at low density, and whose elusiveness includes a great avoidance of humans (Ghimirey & Pal, 2009; Li et al., 2014; Li, McShea, Wang, Gu, et al., 2020; O'Connell et al., 2011). Although most camera‐trapping surveys do not specifically target Asiatic golden cats, detections of the cats as by‐catch during other studies (e.g., Gumal et al., 2014; Li, McShea, Wang, Shen, et al., 2020; Wang et al., 2019) provide us an opportunity to examine their occurrence and distribution status at large scale.

In this study, we collected the state‐wide occurrence records of Asiatic golden cats in China, for the first time, from 2008 to 2019, and all but one record were obtained through camera‐trapping surveys. Based on these data, we constructed a random forest (RF) model to predict its current distribution across the country. Among the four identified regional populations, we further examined the variations of coat morphology in each population. Our results update the distribution status of Asiatic golden cats in China and provide valuable information to guide future research and conservation planning of this elusive and threatened cat.

## 2 METHODS

Using the terms “Asiatic golden cat,” “*Catopuma temminckii*,” the synonym “*Pardofelis temminckii*,” and “China,” in both Chinese and English, we conducted a comprehensive literature search in online databases (i.e., Web of Science [https://webofknowledge.com/], Google Scholar [https://scholar.google.com], Chinese National Knowledge Infrastructure, and Chinese Science and Technology Journal Database), for pertinent peer‐reviewed articles. We limited that search to articles published from 2008 to 2019 because the first camera‐trapping record of the Asiatic golden cat in China was in 2008 (He et al., 2016; Li et al., 2010), and records prior to 2008 are too old to accurately indicate the species' current distribution. That search yielded 12 Asiatic golden cat presence records in 21 publications. Next, we searched Baidu (https://www.baidu.com/) and Google (https://www.google.com/), the most popular search engines in Chinese and English, respectively, for news reports of the Asiatic golden cat, and obtained seven presence records during the study period. The regional camera‐trapping networks managed by the authors (Li, McShea, Wang, Shen, et al., 2020; Liu, Song, et al., 2020; Shen, Li, et al., 2020; Shen, Yu, et al., 2020; Wang et al., 2019) provided another 390 camera detections of Asiatic golden cats with exact geographical coordinates. After deleting duplicate sightings at each camera station, 55 presence sites (all with coordinates from 17 protected areas) of the species were confirmed across China.

We reviewed the baseline survey reports of nature reserves to collect coarse‐resolution absence data of the Asiatic golden cats following the approach proposed by Shen et al. (2021). When a nature reserve is established in China, a baseline survey is required. The survey report usually contains a species list of the reserve based on wildlife records typically compiled since the mid‐20th century (i.e., after the establishment of the P. R. China), since when there have been systematic wildlife investigations across the country (Li, McShea, Wang, Gu, et al., 2020). Although Asiatic golden cat is on the species list in many reserves, we considered this “presence” data were not reliable to indicate its current occurrence since the list may contain historical records, especially given the rapid population decline and habitat loss over the recent decades. We defined an “absence” within a reserve when Asiatic golden cat is not listed in that reserve's baseline survey and decided that the Asiatic golden cat's absences in those surveys were not false‐negatives (Shen et al., 2021). After searching 143 published baseline surveys of national nature reserves in China (e.g., Ye et al., 2014), we obtained 49 absence records.

All presence/absence sites were processed using ArcMap 10.5 (ESRI Inc., 2017) to generate geo‐referenced vector point layers. Given that geographic sampling bias may lead to an over‐representation of environmental variables in high sampling areas (Williams et al., 2002), we used the “spThin” package (Aiello‐Lammens et al., 2015) in R 3.6.1 (R Core Team, 2019) to thin our dataset at a spatial resolution of 30 km. Since many collected sites are coarse records at the reserve level, the 30‐km resolution used in the modeling will match the reserves in scale and largely avoid model overfitting. That spatial thinning reduced redundancy and class imbalance prior to model construction (Breiman, 2001; Cutler et al., 2007) and left a final dataset of 22 presence and 48 absence points (Figure 2).

We collected 25 candidate environmental variables (Appendix 1), all resampled to the 30‐km resolution, and examined the paired correlations between them, retaining the more ecologically meaningful one of each pair of variables that had a correlation coefficient >0.8 (Dormann et al., 2013). Thus, we obtained four climate variables remained: Temperature Seasonality (bio4), Minimal Temperature of Coldest Month (bio6), Mean Temperature of Coldest Quarter (bio11), Precipitation Seasonality (bio15). We also retained other variables: elevation (DEM), terrain ruggedness (DEMstd), human modification (HM) and tree cover (Appendix 1) for subsequent model construction.

We then predicted the species distribution by using the RF algorithm in the “biomod2” package (Breiman, 2001; Thuiller et al., 2016; Wilfried et al., 2009) in R 3.6.1 (R Core Team, 2019). Given our small dataset, we used cross‐validation by five replicates (each replicate used 80% of the data for model training and the remaining 20% for model evaluation), and used ROC (receiver operating characteristic)‐AUC (area under curve) to evaluate the model performance (Fielding & Bell, 1997). When the model evaluation performs well, we choose the model be trained on all available data as final model. In addition to AUC, we used Continuous Boyce Index (CBI) as an additional assessment measure (Boyce et al., 2002; Hirzel et al., 2006). We used variable importance computed by Random Forest algorithm and displayed the response curve of the model (Elith et al., 2005; Thuiller et al., 2016). We converted continuous predicted distribution probability into binary maps using the 10th percentile presence threshold (Guisan et al., 2017). Given our coarse mapping resolution (i.e., 30 km), the model's prediction likely overestimated the range while converting the results to the binary map. To examine the impacts of different cut‐off values on the final maps, we also used higher, alternative cut‐off values (i.e., 0.75, 0.80, 0.85, and 0.90) to generate a series of binary maps as references (Appendix 2). We run distribution models with different resolutions (1, 5, 10, and 30 km) under RF algorithms and also run ensemble model using three algorithms (generalized linear model, gradient boosting machine, RF algorithms) (Appendix 3).

According to both the predicted distribution range and geographical barrier factors, we divided our predicted results into four regions (Shen et al., 2021) and summarized the survey efforts in each of those regions, while considering that each harbors a regional population or meta‐population of Asiatic golden cats. To compare our model prediction to the cat's historical distribution in China, we digitized their historical range as shown in the species' distribution map in *A Guide to the Mammals of China* (Smith & Xie, 2008). We also compared our predicted range with the IUCN Asiatic golden cat range map (https://www.iucnredlist.org/species/4038/97165437; McCarthy et al., 2015).

**FIGURE 1:**
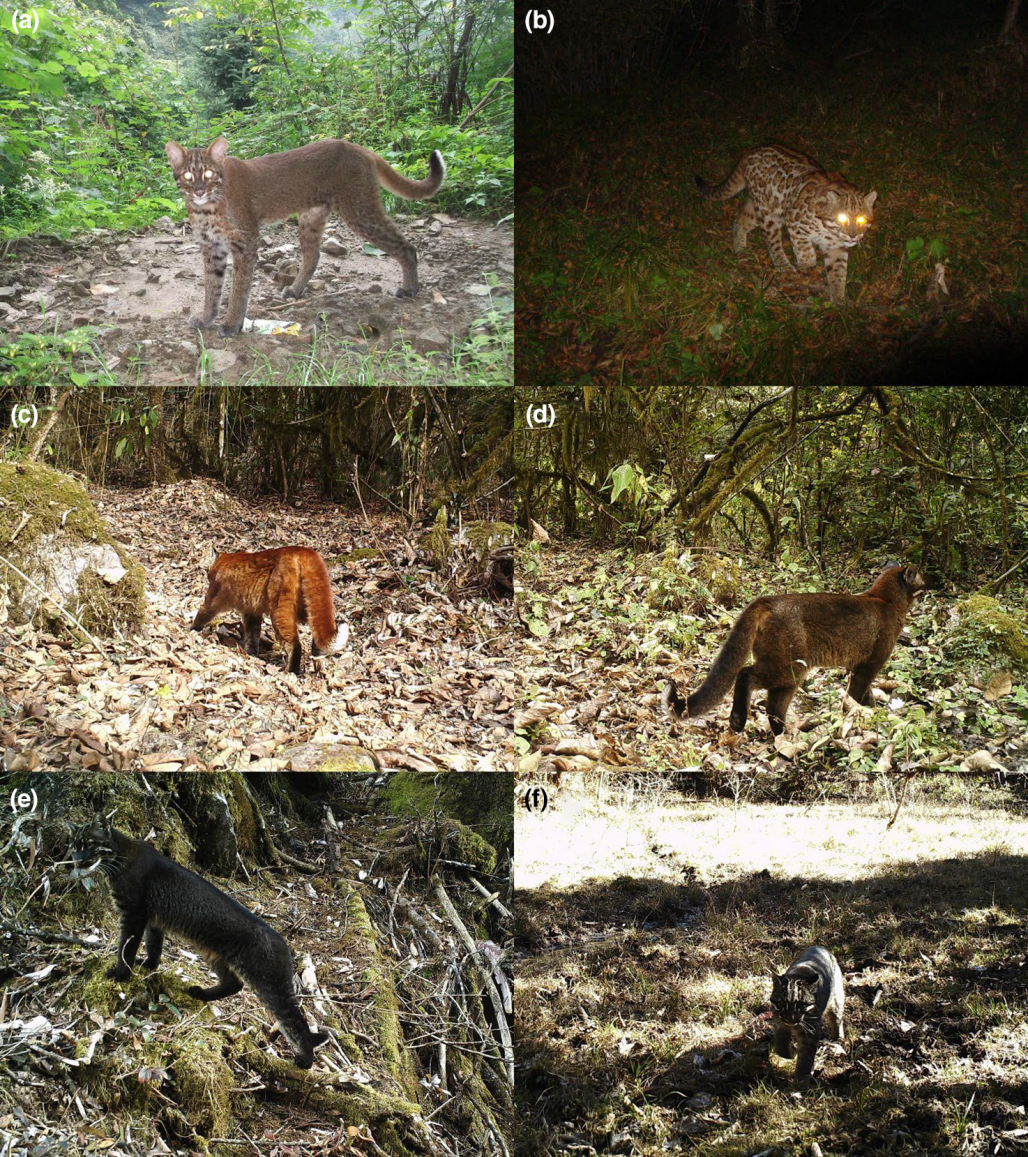
Representative camera‐trap photographs of the six typical coat morphs of Asiatic golden cats in China. (a) Common golden, (b) spotted, (c) red, (d) dark cinnamon, (e) melanistic, (f) gray. Camera locations were in (a) Laohegou Nature Reserve, Sichuan Province; (b) Tangjiahe National Nature Reserve, Sichuan Province; and (c–f) Yarlung Zangbo Grand Canyon National Nature Reserve, Tibet Autonomous Region.

We used photographs and videos to examine the coat morphs of 287 recognizable Asiatic golden cats captured on camera. We followed the coat morph definitions proposed by Wang et al. (2019), but with slight modifications according to previous studies (Gao et al., 1987; Nijhawan et al., 2019; Smith & Xie, 2008; Wilson & Mittermeier, 2014). We classified those coat morphs into six types: (1) The common golden morph (Figure 1a) is reddish‐brown without obvious spots or stripes. It may have blurry dark brown solid spots on the abdomen and four legs, especially visible on the inner side of the legs. (2) The spotted morph's (Figure 1b) grayish‐brown to red pelage is covered with conspicuous rosette spots (black or dark brown edges within light brown or yellow interior) on the flanks and shoulders. It has black bars on the tail; dark, dorsal stripes; and dark, solid spots on the legs. (3) The red morph (Figure 1c) is uniformly bright, fox red to dark reddish‐brown, similar to the coat color of the red muntjac (*Muntiacus vaginalis*), without any spots on the back and flanks. (4) The dark cinnamon morph (Figure 1d) is uniformly dark cinnamon to dark brown without obvious spots on the back and flanks. (5) The melanistic morph (Figure 1e) has a base color of black or dark gray, sometimes with rather blurry rosette spots on the flanks. (6) And the gray morph's (Figure 1f) overall pelage is lead gray, much paler than that of the melanistic morph, and without obvious spots and stripes. The individuals of different color morphs also share some common features (Figure 1). First, their facial markings are relatively consistent, with three darks stripes on the forehead, a wide white stripe at the inner corner of each eye, white eyebrows, and a white stripe on each side of the cheek that runs diagonally from below the eye to below the ear. Next, the white typically found on their jaws varies in width among the different coat morphs, and the backs of their ears are black. Finally, their tail tips are always upturned and are black on the tip of the dorsal side and clearly bright white on the ventral side. We calculated both coat morph percentages and the relative abundance index (RAI), which we defined as the number of independent detections per 100 camera days (Li, McShea, Wang, Gu, et al., 2020; O'Brien, 2011) of each morph within each recognized regional population.

## 3 RESULTS

**FIGURE 2:**
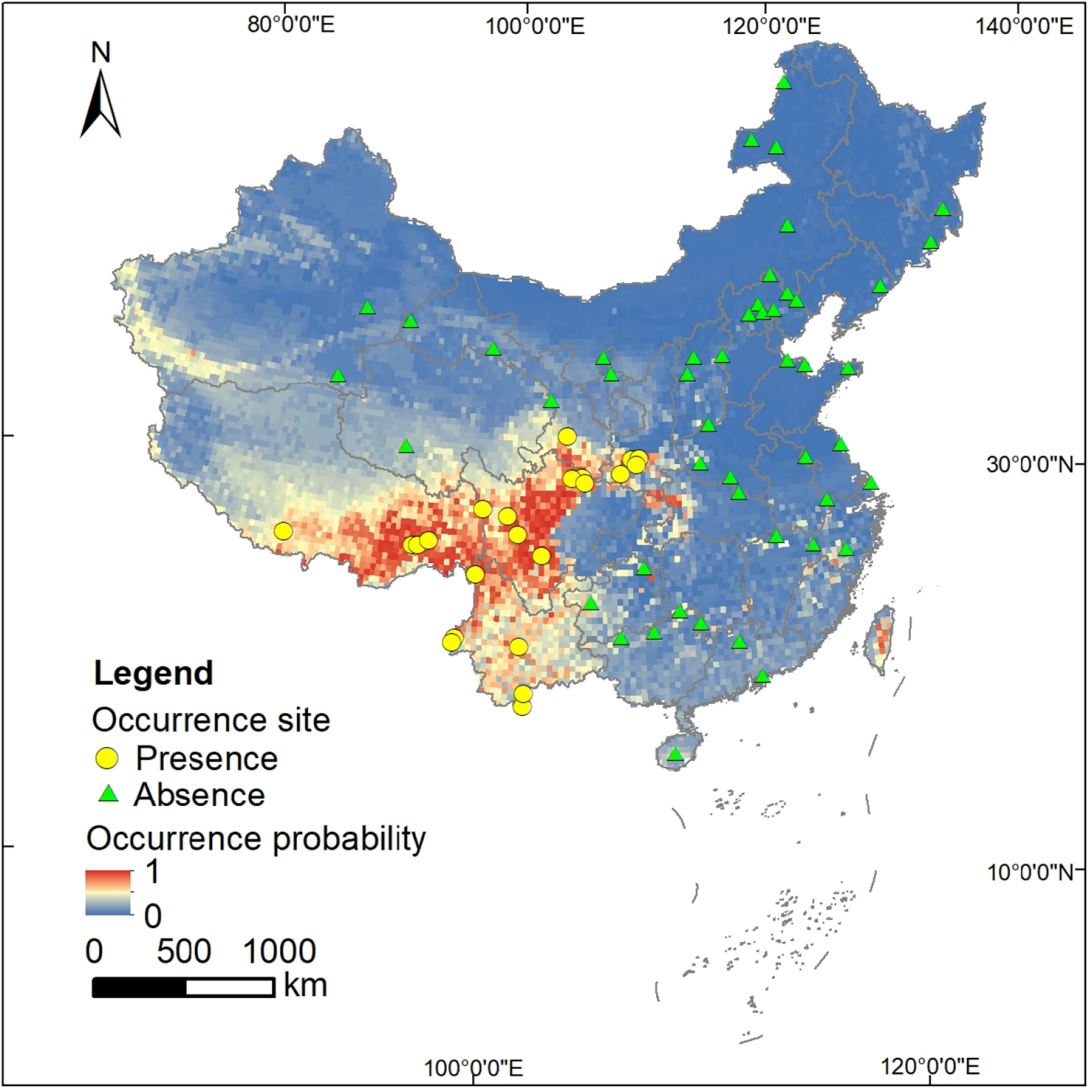
Occurrence points in 30 km × 30 km grids and the predicted occurrence probabilities of the Asiatic golden cat in China.

**FIGURE 3:**
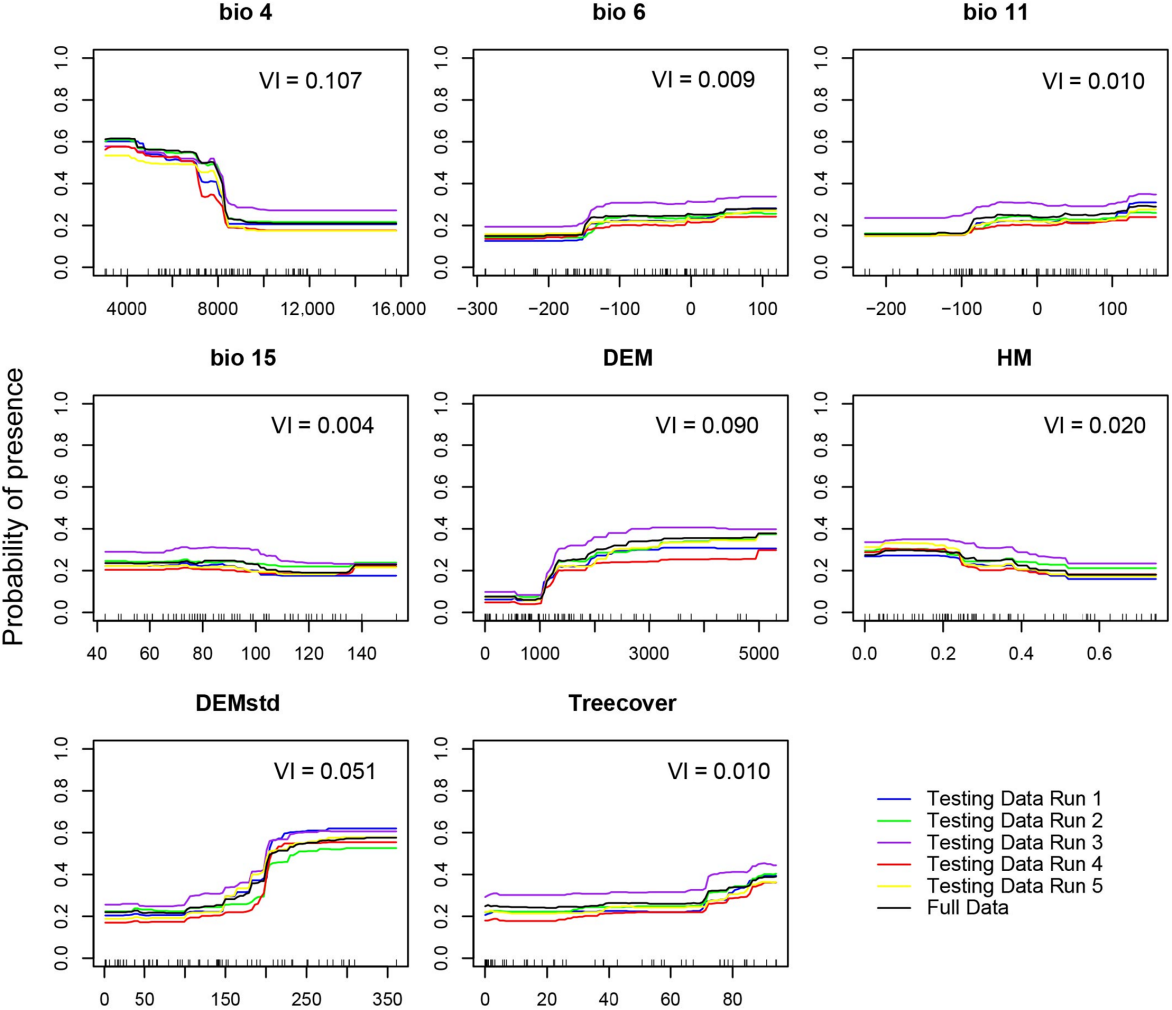
The variable importance (VI) and response curves of the environmental predictors used in the random forest model to predict the current distribution of Asiatic golden cats in China. bio4, temperature seasonality; bio6, Minimal Temperature of Coldest Month; bio11, Mean Temperature of Coldest Quarter; bio15, Precipitation Seasonality; DEM, Elevation; DEMstd, terrain ruggedness; HM, human modification.

The cross‐validation revealed an average AUC was 0.944 and the model based on full training data showed a high CBI of 0.861, indicating a good prediction performance. The predicted range was located mainly in southwestern China, from the Qinling Mountains in southern Shaanxi Province, through the Hengduan Mountains along the eastern edge of the Qinghai‐Tibet Plateau, to the eastern Himalayas and the mountainous areas of western and southern Yunnan Province (i.e., the northern Indo‐China region) (Figure 2). Additionally, the Central Mountains of Taiwan were predicted to be highly suitable Asiatic golden cat habitat, even though the species has not been found on that island (Figure 2). Among the environmental predictors, the three most important predictors were temperature seasonality (bio4), elevation (DEM), and terrain ruggedness (DEMstd) (Figure 3; Appendix 1). Asiatic golden cats prefer to inhabit areas with low seasonal temperature variation, high elevation, and rugged terrain (Figure 3).

**FIGURE 4:**
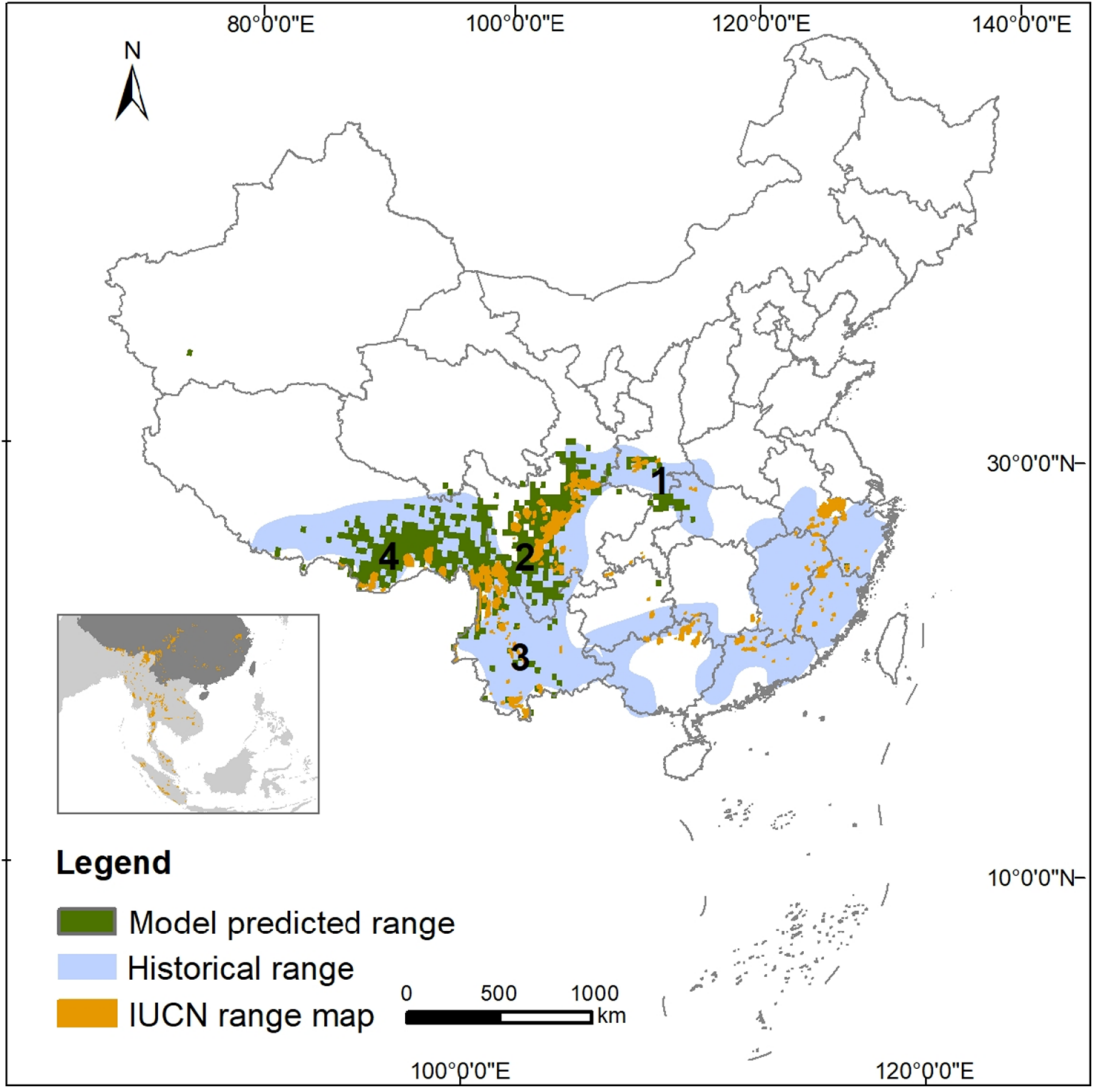
The model predicted current range of Asiatic golden cats in China, compared to the species' historical range and IUCN's range map. The four regions are (1) the Qinling Mountains, (2) the Hengduan Mountains, (3) Southern Yunnan, and (4) the East Himalayas. The small map in the lower left box shows the entire range of Asiatic golden cat in East and Southeast Asia.

The binary map, excluding Taiwan Island, showed an estimated 443,700 km^2^ Asiatic golden cat distribution area in China (Figure 4). The maps using the alternative higher cut‐off values (i.e., 0.75, 0.80, 0.85, and 0.90) had predicted range areas of 414,900, 351,900, 282,600, and 212,400 km^2^, respectively (Appendix 2).

The four regions that we divided the predicted distribution area into were as follows: (1) the Qinling Mountains, which included the Qinling Mountains in southern Shaanxi Province and several nearby mountainous areas in northeastern Sichuan and western Hubei provinces (e.g., the Daba Mountains); (2) the Hengduan Mountains, a series of mountains along the eastern edge of the Qinghai‐Tibet Plateau and located in part of southern Gansu and much of western Sichuan Provinces; (3) Southern Yunnan, a mountainous region from central to southern Yunnan Province and connected to the species' range in Southeast Asia (e.g., Laos); and (4) the East Himalayas, which included southeastern Tibet and part of northwestern and western Yunnan Province and connected to its range in India and Myanmar (Figure 4). Among those four distribution regions (surveyed at a resolution of 30 km), two of them, the Hengduan Mountains and East Himalayas, appear to be a continuous patch, but they are actually separated by the deep, dry valleys created by the Yangtze River. Similarly, we considered the Hengduan Mountain region separated from the Southern Yunnan region by intensive agriculture and natural geographic barriers in central Yunnan Province.

**FIGURE 5:**
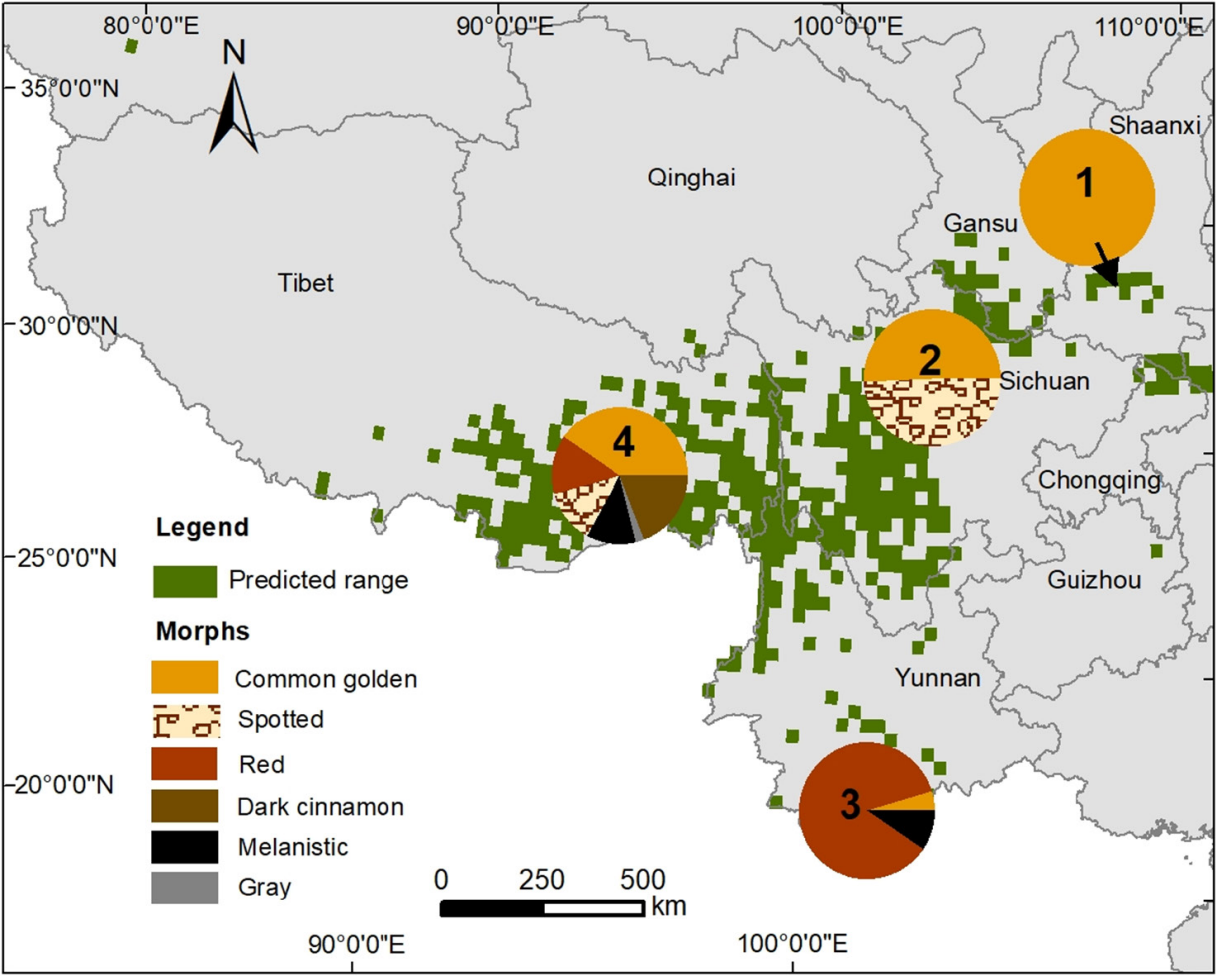
The proportions of independent detections of each coat morph of Asiatic golden cats in each regional population in China. (1) The Qinling Mountains (*n* = 32), (2) the Hengduan Mountains (*n* = 83), (3) Southern Yunnan (*n* = 21); and (4) the East Himalayas (*n* = 151).

Among the 390 Asiatic golden cat camera‐trapping detections (32, 88, 21, and 249 from the Qinling Mountains, Hengduan Mountains, Southern Yunnan, and East Himalayas, respectively), the specific coat morphs of 287 adults were identified. Among the 287 recognizable detections, there were 47.4% common golden, 20.9% spotted, 13.6% red, 10.1% dark cinnamon, 7.0% melanistic, and 1.0% gray morphs (Table 1). Only the common golden morph was recorded in the Qinling Mountains and two coat morphs, common golden and spotted, were recorded in similar proportions (50.6% and 49.4%, respectively) in the Hengduan Mountains. Three coat morphs, common golden (4.8%), red (85.7%), and melanistic (9.5%), were recorded in Southern Yunnan, and the East Himalayas had all six coat morphs (common golden 40.4%, spotted 12.6%, red 14.0%, dark cinnamon 19.2%, melanistic 11.9%, and gray 2.0%), as well as and the highest overall abundance of detections (RAI = 0.578) (Table 1). The composition and complexity of coat morphs within the regional populations increased in a northeast to southwest gradient (Figure 5).

**TABLE 1:** The coat morphs and relative abundances of Asiatic golden cats in the four regional populations in China.

Region (survey efforts^a^/overall RAI^b^)	Coat morph	No. of independent detections	RAI^b^
1. Qinling Mts. (243,159/0.013)	Common golden	32	0.013
2. Hengduan Mts. (28,623/0.290)	Common golden	42	0.147
	Spotted	41	0.143
3. S Yunnan (23,405/0.090)	Common golden	1	0.004
	Red	18	0.077
	Melanistic	2	0.009
4. E Himalayas (26,135/0.578)	Common golden	61	0.233
	Spotted	19	0.073
	Red	21	0.080
	Dark cinnamon	29	0.111
	Melanistic	18	0.069
	Gray	3	0.011

^a^ Measured as the total number of camera days accumulated in camera‐trapping surveys across the region.

^b^ RAI, relative abundance index defined as the number of independent detections per 100 camera days. RAI = No. of independent detections × 100/No. of camera days.

## 4 DISCUSSION

All but one (i.e., taken by the staff of Tangjiahe Reserve with a hand‐held camera in 2019) of our Asiatic golden cat detections were from the camera‐trapping surveys (e.g., Li, McShea, Wang, Shen, et al., 2020; Wan et al., 2020), including those reported by the news. Over the past two decades, a series of regional or state‐wide camera‐trap monitoring networks, including over 50,000 cameras, have been established in China (Li, 2020; Li et al., 2018; Xiao et al., 2014), generating an increasing collection of Asiatic golden cat detections as by‐catch records. Although this study did not obtain all possible records, we still believe it a good example of how combining the data from different sources to determine the natural history and ecology of an elusive species. We thus advocate that to facilitate the research of wide‐ranging species, different monitoring networks, both within a country and between countries, should facilitate data sharing in the spirit of cooperative research practices (McShea et al., 2020).

Using the species distribution model, we were able to utilize the best available data to predict the current distribution of Asiatic golden cat in China. Our results showed that the distribution of Asiatic golden cat was mainly determined by climate and topography. The importance of environment variables may vary at different resolutions (Shen et al., 2021). In the future, studies with different precision are needed to fully understand the effects of different variables. Our predicted range is within the historical range of the species, expect for a few scattered pixels, and it shows the complete disappearance of cat populations in the vast eastern and southern regions of China. Our predicted results may underestimate the probability of species distribution in southern Yunnan. Compared to the IUCN range map (McCarthy et al., 2015), our model's predicted range extended farther north, to southern Gansu Province, and that agrees with recent records from that region (L. Zhu, unpublished data). However, unlike the IUCN range map, ours indicates the possible extirpation of this species from central, eastern, and southern China. Once widely distributed across this vast area, no confirmed records of Asiatic golden cats have been reported since 2008, despite tremendous camera‐trapping efforts within the area (e.g., Li et al., 2018; Shen, Yu, et al., 2020; Wan et al., 2020). The rather low detection rates of Asiatic golden cats within its current range also indicated that the threatened status of this species in China is severe and there is an urgent need for prioritized consideration and specific conservation actions. Due to the limited data size, our current research can only produce a coarse‐resolution result. In the future, with rapidly increasing accumulation of camera‐trapping monitoring data across the country (Li, 2020), the species distribution model will be more accurate in predicting the distribution area of Asiatic golden cat at finer resolutions, which will better support further conservation activities on this endangered species.

Additionally, our predicted range in southwest China is much larger than the IUCN range, although the resolution of the expert knowledge‐based IUCN map appeared to be finer than ours. It is possible that our model, using the 0.731 threshold, overestimated the potential distribution, especially given the coarse modeling resolution. We chose10th percentile presence threshold to simulate most of the observed presences and to assure that most simulated presences were consistent with observed presences (Liu et al., 2005). But, the binary map generated at an alternative higher threshold (0.80) (Appendix 2) agreed better with the expert knowledge‐based IUCN map. For future studies, besides enlarging the sample size to ensure a more detailed distribution modeling at finer spatial resolution, we also suggest that expert opinion be involved at the modeling stage to determine an appropriate threshold. That means that an integrative mapping approach that combines expert knowledge and species distribution modeling may improve the accuracy of the final map. We performed models at different resolutions (1, 5, 10, and 30 km), but due to limited data, the high‐resolution results did not perform as well as the low‐resolution ones (Appendix 3). At a resolution of 30 km, we used an ensemble model approach, and the predicted results overestimated the probability of species distribution in Xinjiang (Appendix 3).

Among the four regional populations of Asiatic golden cats, the Qinling and Hengduan Mountain populations were entirely within China, and both are sheltered by regional protected area networks formed by adjacent nature reserves (Hu et al., 2018; Li, McShea, Wang, Gu, et al., 2020; Shi et al., 2017). The other two populations were transboundary. For many threatened species, transboundary populations are especially vulnerable, due in large part to excessive poaching pressure and poor law enforcement against illegal wildlife trade along the political boundaries (Liu, Yong, et al., 2020; Nijman & Shepherd, 2015; Vitkalova et al., 2018). In particular, the transboundary Asiatic golden cat populations between China and Laos and Myanmar (i.e., the Southern Yunnan population) requires immediate attention because of its rather low abundance (overall RAI = 0.090).

We showed that the Asiatic golden cat's coat morph composition and complexity within the four regional populations in China increased on a northeast to southwest gradient, and that the East Himalayas population had the most coat morph diversity. Previous studies in Bhutan (Dhendup et al., 2016; Jigme, 2011), Nepal (Ghimirey & Pal, 2009), India (Choudhury, 2007; Nijhawan et al., 2019), and China (Feng et al., 1986; Wang et al., 2019) have indicated that the Asiatic golden cat population in the tropical montane forests of the Central to East Himalayas are highly morphologically diverse. Of the six coat morphs we recorded in that region, four were previously reported by Wang et al. (2019), but the spotted and dark cinnamon morphs are new to southeastern Tibet. The causes underlying that diversity could be related to a higher degree of both habitat fragmentation and human pressure in the northeast mountain areas, which could have led to an abrupt decline of local populations accompanied by a loss of genetic diversity and coat morphology. Further work examining that hypothesis should involve population genetics studies that compare both museum specimens and current wild populations.

The ecological mechanisms underlying the polymorphism in Asiatic golden cats have not been fully examined. Color polymorphism and/or the co‐occurrence of different color morphs in a region is a conspicuous feature of many species and a lens through which to explore broad ecological and evolutionary questions, such as the effects of predation and mating systems. Also, it may accelerate speciation and promote sympatric ecological and evolutionary diversification (Gray & McKinnon, 2007; Hugall & Stuart‐Fox, 2012; McKinnon & Pierotti, 2010). Forsman et al. (2008) summarized the effects of animal color patterns on fitness and concluded that coat polymorphism may confer multiple selective advantages, such as greater colonization and range expansion successes, and better camouflage (Schneider et al., 2015). Individuals of the melanistic and gray morphs of the Asiatic golden cat may consider ecologically adapted to a wide range of ecological niches (Nijhawan et al., 2019). We hypothesize that polymorphism in Asiatic golden cats may broaden the species' spatial and temporal niches and thus reduce intra‐guild competition and predation. Undoubtedly, the Asiatic golden cat can serve as an ideal model for vertebrate polymorphism studies that investigate the ecological adaptations behind diverse coat morphology.

Patel et al. (2016) combined a gradual variation of pelage pattern, molecular data, and species distribution projections to explain evolutionary processes of Asiatic golden cat and support the hypothesis of a post‐Toba population expansion of the Asiatic golden cat from south China/Indo‐China to Peninsular Malaysia and Sumatra. For future studies, we propose to incorporate more historical geographic environmental variables (e.g., geographic distances from inferred glacial refugia) to explain evolutionary processes (Darul et al., 2022). Furthermore, Xu et al.'s (2017) work on tigers identified multiple genes (e.g., *CORIN* and *SLC45A2*) with key roles in the formation of hair pigmentation and color variants (i.e., normal, white, golden, and unstriped snow white). Besides the six coat morphs we defined in this study, we also noticed Asiatic golden cat individuals with varying degrees of intermediate morphological characteristics. Nijhawan et al. (2019) also observed intermediate morphs, such as the “compact rosy” morph, in the Eastern Himalayan Asiatic golden cat population. The abundant polymorphism in Asiatic golden cats indicates that this species' complicated genetic mechanisms and evolutionary developmental progression needs further exploration.

Large carnivores (e.g., tigers, leopards, and dholes *Cuon alpinus*) have been extirpated in some areas, such as the Minshan and Xiangling Mountains in Sichuan Province (Li, McShea, Wang, Gu, et al., 2020; Li, McShea, Wang, Shen, et al., 2020), that are within the Asiatic golden cat distribution range in China. That left the Asiatic golden cat the largest felid, and perhaps the top predator, in these areas. Meso‐carnivore release is a common consequence following the loss of large carnivores in terrestrial ecosystems (Crooks & Soulé, 1999; Ritchie & Johnson, 2009), but due to the lack of long‐term monitoring of Asiatic golden cat population dynamics in China, this phenomenon has not been examined and reported in these regions. Although typically considered a small cat and a meso‐carnivore in forest ecosystems, adult Asiatic golden cats, with a maximum body weight exceeding 15 kg (Liu, Wu, et al., 2020), are capable of killing large prey such as ungulates (e.g., wild boar *Sus scrofa*, Chinese goral *Naemorhedus griseus*, and forest musk deer *Moschus berezovskii*) and primates (e.g., Rhesus macaque *Macaca mulatta* and golden snub‐nosed monkey *Rhinopithecus roxellana*) (Xiong et al., 2017). Future studies in such degraded ecosystems should focus on the role and functions of the Asiatic golden cat in the system's trophic structure and ecological processes.

## AUTHOR CONTRIBUTIONS

**Fei Duan:** Data curation (equal); formal analysis (lead); methodology (lead); visualization (lead); writing – original draft (lead); writing – review and editing (equal). **Shuyi Zhu:** Data curation (equal); methodology (supporting); validation (supporting); visualization (supporting); writing – review and editing (supporting). **Yuan Wang:** Data curation (equal); investigation (equal); validation (equal); writing – review and editing (supporting). **Dazhao Song:** Data curation (equal); investigation (equal); validation (equal); writing – review and editing (supporting). **Xiaoli Shen:** Conceptualization (lead); project administration (supporting); supervision (supporting); validation (equal); writing – review and editing (supporting). **Sheng Li:** Conceptualization (lead); data curation (equal); funding acquisition (lead); investigation (equal); methodology (equal); project administration (lead); resources (lead); supervision (lead); validation (equal); writing – review and editing (equal).

## CONFLICT OF INTEREST STATEMENT

None.

## Data Availability

The data that support the findings of this study are available in figshare at DOI: 10.6084/m9.figshare.24083364.

## APPENDIX 1

### The environmental predictors used in the distribution modeling of the Asiatic golden cat in China

**Table:**

Name	Abbreviation	Resolution	Source
Climate			CHELSA
Annual mean temperature	bio1	1 km	https://chelsa‐climate.org/
Mean diurnal range	bio2	1 km	CHELSA
Isothermality	bio3	1 km	CHELSA
Temperature seasonality^a^	bio4	1 km	CHELSA
Maximal temperature of warmest month	bio5	1 km	CHELSA
Minimal temperature of coldest month^a^	bio6	1 km	CHELSA
Temperature annual range	bio7	1 km	CHELSA
Mean temperature of wettest quarter	bio8	1 km	CHELSA
Mean temperature of driest quarter	bio9	1 km	CHELSA
Mean temperature of warmest quarter	bio10	1 km	CHELSA
Mean temperature of coldest quarter^a^	bio11	1 km	CHELSA
Annual precipitation	bio12	1 km	CHELSA
Precipitation of wettest month	bio13	1 km	CHELSA
Precipitation of driest month	bio14	1 km	CHELSA
Precipitation seasonality^a^	bio15	1 km	CHELSA
Precipitation of wettest quarter	bio16	1 km	CHELSA
Precipitation of driest quarter	bio17	1 km	CHELSA
Precipitation of warmest quarter	bio18	1 km	CHELSA
Precipitation of coldest quarter	bio19	1 km	CHELSA
Topology			
Elevation^a^	DEM	90 m	SRTM (http://srtm.csi.cgiar.org/SELECTION/inputCoord.asp)
Terrain ruggedness^a^	DEMstd	90 m	Standard Deviation of dem
Land cover			
Normalized difference vegetation index	NDVI	500 m	MODIS (http://www.gscloud.cn/; https://modis.gsfc.nasa.gov/data/dataprod/mod13.php)
Tree cover^a^		1 km	Global Forest Watch (https://www.globalforestwatch.org/)
Anthropogenic impacts			
Human modification^a^	HM	1 km	http://gdra‐tnc.org/current/
Human population density		1 km	WorldPop (https://www.worldpop.org)

^a^ These variables were used in the final model.

## APPENDIX 2

The predicted ranges of Asiatic golden cats shown in binary maps generated with alternative cut‐off values: (a) 0.75, (b) 0.80, (c) 0.85, (d) 0.90.

## APPENDIX 3

The occurrence probability of Asiatic golden cats in China derived from different models at different resolutions. (a) Random forest model with 1‐km resolution, (b) random forest model with 5‐km resolution, (c) random forest model with 10‐km resolution, (d) ensemble model with 30‐km resolution.
